# MRI-based habitat radiomics for preoperative prediction of axillary pathological complete response in breast cancer after neoadjuvant therapy: a multicenter study

**DOI:** 10.3389/fonc.2026.1801530

**Published:** 2026-05-13

**Authors:** Lei Ma, Shunian Li, Ziwei Cao, Jun Liao, Mengting Xu, Chuanjian Lv, Chunmiao Xu, Hongna Tan

**Affiliations:** 1 Department of Radiology, Henan Provincial People’s Hospital, Zhengzhou, China; 2 Department of Radiology, People’s Hospital of Zhengzhou University & Henan Provincial People’s Hospital, Zhengzhou, China; 3 Department of Radiology, People’s Hospital of Henan University & Henan Provincial People’s Hospital, Zhengzhou, China; 4 Department of Medical Imaging, The Affiliated Cancer Hospital of Zhengzhou University & Henan Cancer Hospital, Zhengzhou, China

**Keywords:** axillary pathological complete response, breast cancer, habitat radiomics, magnetic resonance imaging, neoadjuvant therapy

## Abstract

**Purpose:**

To develop and validate a preoperative MRI-based habitat radiomics nomogram for noninvasive prediction of axillary pathological complete response (apCR) after neoadjuvant therapy (NAT) in node-positive breast cancer.

**Patients and methods:**

This retrospective multicenter study included patients with histologically confirmed node-positive breast cancer from two institutions who underwent pretreatment breast MRI. Dynamic contrast-enhanced MRI was used for tumor segmentation and to define intratumoral habitat subregions based on enhancement heterogeneity. Radiomic features were extracted from whole tumors and habitat subregions, and radiomics and habitat signatures were integrated with clinicopathologic variables to construct a nomogram for preoperative prediction of apCR. Model performance was evaluated using receiver operating characteristic (ROC) analysis, calibration assessment, and decision curve analysis (DCA).

**Results:**

A total of 336 women were included. In the training cohort, the radiomics, habitat, and nomogram models achieved AUCs of 0.723, 0.765, and 0.845, respectively. The nomogram consistently demonstrated the highest discriminative performance in the internal validation and independent external test cohorts, with AUCs of 0.755 and 0.754. Calibration analysis showed good agreement between predicted and observed apCR, and DCA indicated greater net clinical benefit for the nomogram.

**Conclusion:**

The proposed MRI-based habitat radiomics nomogram showed promising performance for noninvasive, preoperative prediction of apCR after NAT and may assist individualized axillary risk stratification in patients with node-positive breast cancer.

## 1 Introduction

Breast cancer is the most commonly diagnosed malignancy among women worldwide and remains a leading cause of cancer-related mortality (1, 2). In clinical practice, axillary lymph node (ALN) status is a critical determinant of prognosis and plays a central role in guiding decisions regarding axillary surgical management and adjuvant treatment strategies (3). With the increasing adoption of neoadjuvant therapy (NAT) in patients presenting with initially node-positive disease, a substantial proportion of patients achieve axillary pathologic complete response (apCR). Reported apCR rates range from 40% to 75% (4–6). This finding has prompted interest in de-escalating axillary surgery in selected patients (7). Accordingly, accurate post-NAT assessment of residual ALN disease plays a key role in guiding personalized treatment strategies and prognostic evaluation. Although axillary lymph node dissection (ALND) remains the reference standard for pathological evaluation of residual nodal disease and assessment of apCR, it is invasive and associated with substantial morbidity, including pain, lymphedema, and nerve injury (8). Less invasive approaches, such as sentinel lymph node biopsy and targeted axillary dissection, have been introduced to reduce surgical burden. However, their clinical utility is limited by false-negative rates and the continued need for operative intervention (9–11). Consequently, as nodal downstaging after NAT becomes more common, accurate and noninvasive tools are needed to reliably identify residual ALN disease.

MRI provides superior soft-tissue contrast and functional information for the assessment of treatment response after NAT. Radiomics has emerged as a quantitative imaging approach that extracts high-dimensional features from medical images and may capture information related to underlying tumor biology and clinical phenotypes (12, 13). Most MRI-based radiomics studies, however, rely on features derived from the whole tumor volume (14–16). This approach may overlook spatial heterogeneity within tumors, which often show marked regional differences in vascularity, cellularity, and stromal composition, particularly after systemic therapy (17–19).

Habitat radiomics addresses this limitation by subdividing tumors into spatially distinct subregions with different imaging phenotypes (20, 21). Dynamic contrast-enhanced MRI (DCE-MRI) is well suited for this purpose, as enhancement patterns reflect tumor perfusion and vascular permeability, which are closely associated with treatment response and metastatic potential (22, 23). In this study, we developed and validated a DCE-MRI–based habitat radiomics model integrating clinical variables, conventional radiomic features, and habitat-derived heterogeneity metrics to predict post-NAT ALN status. We hypothesized that capturing intratumoral enhancement heterogeneity would improve predictive performance beyond traditional radiomics and clinical factors, thereby supporting individualized axillary surgical decision-making in node-positive breast cancer.

## 2 Materials and methods

### 2.1 Study population

This retrospective multicenter study was approved by the Institutional Review Boards of the participating institutions, and the requirement for informed consent was waived.

**Figure 1:**
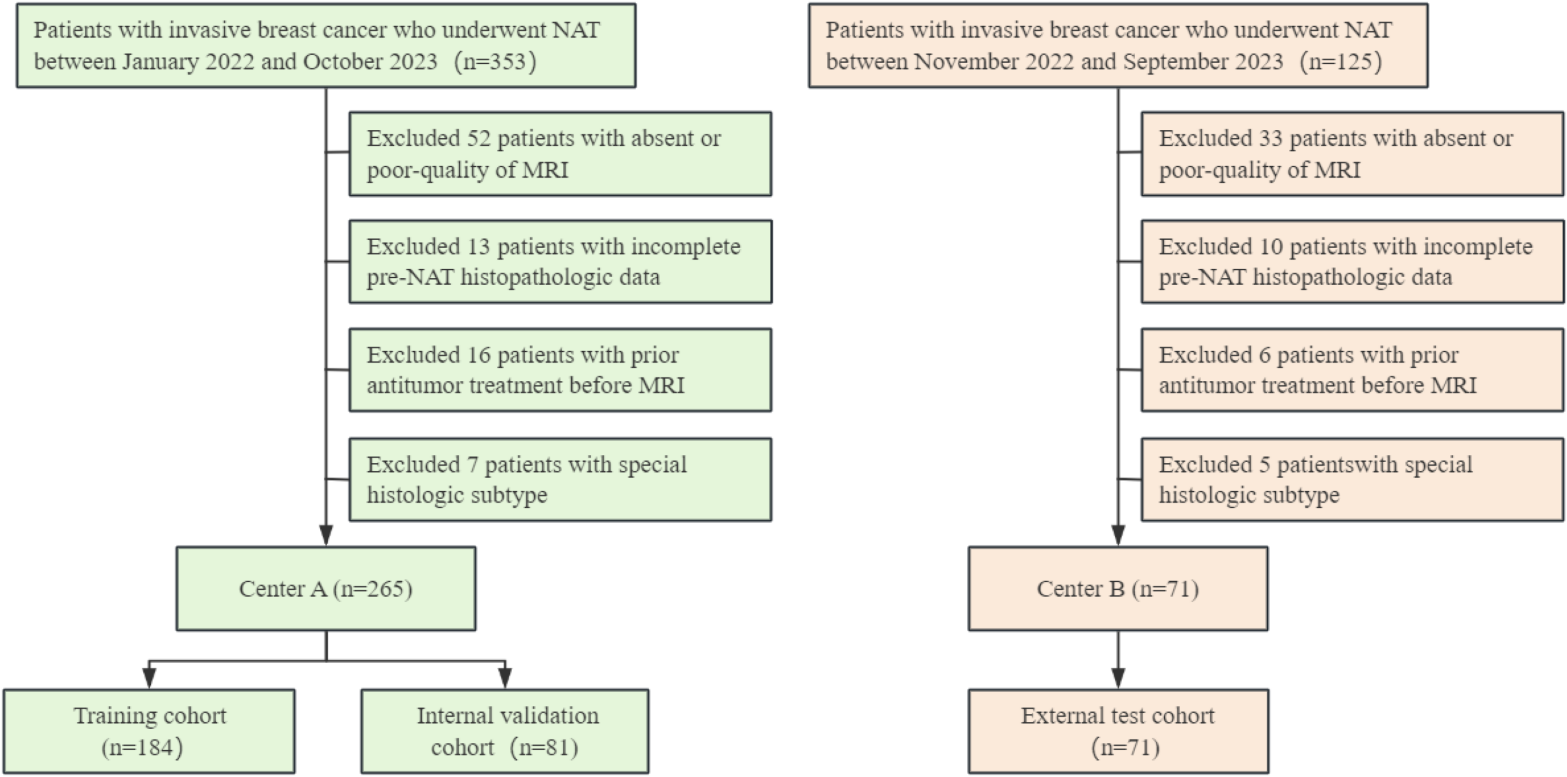
Flowchart illustrating the patient enrollment process for the study.

Consecutive patients were recruited from Henan Provincial People’s Hospital (Center A) between January 2022 and October 2023 and from Henan Cancer Hospital (Center B) between November 2022 and September 2023. Patients from Center A constituted the primary cohort and were used for model development and internal validation, whereas patients from Center B were enrolled using identical criteria and served as an independent external test cohort. The inclusion criteria were as follows: (a) histopathologically confirmed invasive breast cancer with biopsy-proven ipsilateral axillary lymph node metastasis before neoadjuvant therapy (NAT), (b) available pretreatment MRI performed within 2 weeks before NAT, and (c) postoperative pathologic evaluation of treatment response to NAT. Exclusion criteria were: (a) incomplete or poor-quality MRI, (b) receipt of any antitumor treatment, including radiotherapy or chemotherapy, before MRI, (c) incomplete NAT, and (d) special histologic subtype or incomplete pre-NAT histopathologic data. The enrollment process and cohort allocation are summarized in **Figure 1**.

### 2.2 Clinicopathological data collection

Clinicopathological data were retrieved from electronic medical records, including patient age, tumor size, menopausal status, and biomarker status for estrogen receptor (ER), progesterone receptor (PR), human epidermal growth factor receptor 2 (HER2), and Ki-67. Pathological biomarkers were assessed using standard immunohistochemical methods. Hormone receptor (HR) status was defined as positive if either ER or PR was positive. Detailed criteria for biomarker interpretation and molecular subtype classification are provided in **Supplementary Material S1**. Axillary pathological complete response (apCR) was defined as the absence of residual tumor cells in axillary lymph nodes on postoperative pathological examination, whereas any residual nodal disease was classified as non-apCR.

All patients received 6 or 8 cycles of NAT consisting of taxane-based regimens, with or without anthracyclines. Patients with HER2-positive tumors additionally received anti-HER2 targeted therapy. Following completion of systemic treatment, patients underwent either breast-conserving surgery or mastectomy.

### 2.3 Image acquisition

**Figure 2:**
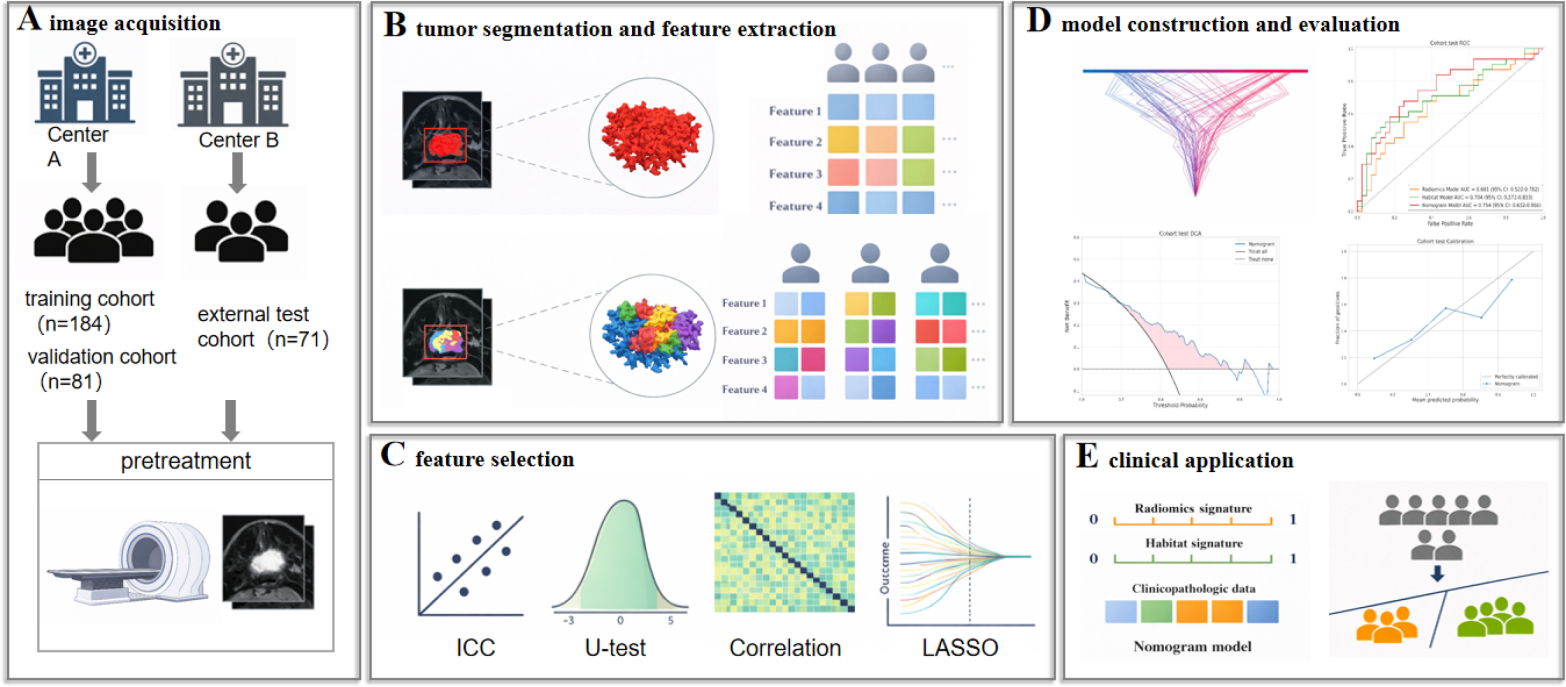
Study workflow. **(A)** image acquisition; **(B)** tumor segmentation and feature extraction; **(C)** feature selection; **(D)** model construction and evaluation; **(E)** clinical application. ICC, intraclass correlation coefficients; LASSO, least absolute shrinkage and selection operator.

All patients underwent baseline breast MRI using dedicated bilateral breast coils on 1.5-T or 3.0-T scanners across the participating centers (**Figure 2A**). Dynamic contrast-enhanced MRI (DCE-MRI) was performed with multiple postcontrast phases acquired according to routine clinical protocols. For radiomic analysis, the third postcontrast phase was selected, as it provided relatively stable lesion enhancement and sufficient contrast for reliable feature extraction. Baseline acquisition parameters, including original in-plane resolution, slice thickness, and scanner-specific imaging settings for each center, are summarized in **Supplementary Table 1**.

### 2.4 Preprocessing and tumor segmentation

Image preprocessing was performed using Python (version 3.11.7). Given that MRI examinations were acquired on 1.5-T and 3.0-T scanners across two institutions, N4 bias field correction was applied to reduce intensity nonuniformity, followed by resampling to an isotropic voxel size of 1 × 1 × 1 mm³ using B-spline interpolation to improve spatial comparability. These preprocessing procedures were performed before radiomic feature extraction to enhance feature reproducibility.

Three-dimensional tumor segmentation was conducted using ITK-SNAP (version 4.2.0). The entire enhancing tumor volume was manually delineated on the third post-contrast DCE-MRI phase by an experienced breast radiologist (L.M.), with careful exclusion of necrotic or hemorrhagic regions, peritumoral edema, and adjacent normal breast parenchyma (**Figure 2B**).

To evaluate inter-observer reproducibility, a randomly selected subset of 33 breast MRI examinations from the full cohort was independently segmented by two radiologists with 8 and 12 years of experience in breast imaging, respectively. Radiomic features were extracted from both sets of segmentations using the same preprocessing and feature-extraction pipeline. Feature-level reproducibility was assessed using intraclass correlation coefficients (ICCs) based on a two-way random-effects model for absolute agreement. ICCs were calculated for all extracted features, and features with ICC values ≥ 0.80 were considered reproducible and retained for further analysis. After reproducibility filtering, 611 whole-tumor radiomic features and 1,302 habitat-derived features were retained for downstream feature selection.

### 2.5 Radiomic feature extraction

Radiomic features were extracted from segmented tumor volumes on DCE-MRI using the PyRadiomics package (version 2.1.1) with default parameter settings (**Figure 2B**). A total of 1,197 quantitative features were generated per tumor, including first-order statistics, shape features, and texture features derived from commonly used matrices, such as the gray-level co-occurrence matrix (GLCM), gray-level dependence matrix (GLDM), gray-level run-length matrix (GLRLM), gray-level size-zone matrix (GLSZM), and neighboring gray-tone difference matrix (NGTDM).

### 2.6 Habitat analysis

Habitat analysis was performed to characterize intratumoral enhancement heterogeneity on DCE-MRI. For each tumor, voxel-wise signal intensities within the segmented tumor volume were first normalized, and unsupervised K-means clustering was then applied independently within each tumor ROI to partition voxels into subregions with similar enhancement patterns (**Figure 2B**). The optimal number of clusters was determined in the training cohort using the Calinski–Harabasz index for candidate values ranging from 2 to 5. A fixed cluster number of five was subsequently adopted for all tumors to ensure consistent habitat definition across patients and cohorts (**Supplementary Table 2**). Because clustering was based on voxel intensity alone and was not spatially constrained, voxels assigned to the same habitat were not required to be spatially contiguous.

Voxels assigned to the same cluster were defined as one imaging habitat, and a corresponding habitat label map was generated for each tumor. Radiomic features were then extracted from each habitat subregion using the same PyRadiomics workflow described above. This yielded 1,197 features per habitat and 5,985 habitat-based radiomic features per patient, which were entered into subsequent model development.

### 2.7 Feature selection

A total of 1,197 whole-tumor radiomic features, 5,985 habitat-based radiomic features, and 10 clinical variables were initially considered. Radiomic features were standardized using z-score normalization, with the mean and standard deviation estimated exclusively from the training cohort and then applied unchanged to the internal validation and independent external test cohorts, thereby avoiding data leakage. To limit overfitting, feature selection and model development were conducted exclusively within the training cohort, with no access to validation or test data during model construction.

Clinical variables were first screened using univariable logistic regression analysis, and variables with P < 0.10 were subsequently entered into multivariable logistic regression to identify independent clinical predictors. In addition, HER2 status was prespecified *a priori* for forced entry into the multivariable analysis because of its established biological and clinical relevance to treatment response in breast cancer, particularly in the neoadjuvant treatment.

Feature reduction for radiomic and habitat-based variables was performed using a multistep feature selection strategy that combined statistical filtering and multivariate selection (**Figure 2C**). Features showing limited discriminative ability were first removed using the Mann-Whitney U test. Redundant features were then excluded based on Spearman correlation analysis, using a correlation threshold of |r| > 0.90. The remaining features were further refined using minimum redundancy–maximum relevance (mRMR), followed by least absolute shrinkage and selection operator (LASSO) regression with 10-fold cross-validation to identify the final feature sets. The numbers of retained features after each selection step, including ICC, Mann–Whitney U, Spearman correlation, mRMR, and LASSO selection, are summarized in **Supplementary Table 3**.

### 2.8 Model development and validation

Using the selected features, two Gaussian naïve Bayes (GNB) classifiers were developed to predict apCR (**Figure 2D**). One classifier was based on whole-tumor radiomic features (radiomics model), and the other was based on habitat-derived features (habitat model). GNB was selected as the base classifier because it is well suited to relatively small-sample, reduced high-dimensional datasets after feature selection, requires limited parameter tuning, and provides probabilistic outputs that can be directly used as compact radiomics and habitat signatures for downstream integration. A combined nomogram was then constructed using multivariable logistic regression by integrating the radiomics signature, habitat signature, and selected clinical predictors (**Figure 2E**), given the interpretability and clinical familiarity of logistic regression for multimodal risk integration.

Model performance was evaluated for the radiomics, habitat, and combined nomogram models across the training cohort, internal validation cohort, and independent external test cohort. Discriminative performance was assessed using the area under the receiver operating characteristic curve (AUC), with sensitivity, specificity, positive predictive value, and negative predictive value reported with 95% confidence intervals. AUCs were compared using the DeLong test. Calibration was evaluated using calibration curves, and clinical utility was assessed using decision curve analysis. The optimal probability cutoff was determined in the training cohort using the Youden index and applied unchanged to the internal validation and external test cohorts.

To evaluate potential overfitting, bootstrap-based optimism correction with 1,000 resamples was performed in the training cohort for the radiomics model, habitat model, and combined nomogram. Optimism was calculated as the mean difference in AUC between each bootstrap sample and the original training cohort, and the optimism-corrected AUC was obtained by subtracting the mean optimism from the apparent AUC. The combined nomogram comprised four predictors, corresponding to an events-per-variable ratio of 19.75 based on 79 apCR events in the training cohort.

### 2.9 Statistical analysis

Statistical analyses were performed using R (version 4.0.5) and Python (version 3.11.7), with SPSS (version 26.0) used for supplementary analyses. Continuous variables were compared using the Student’s t test or Wilcoxon rank-sum test, as appropriate, while categorical variables were analyzed using the χ² test or Fisher’s exact test. Inter-reader reproducibility for tumor segmentation was assessed using the ICC based on a two-way random-effects model. All statistical tests were two-sided, and P < 0.05 was considered statistically significant.

## 3 Results

### 3.1 Characteristics of patients

A total of 336 women with biopsy-proven invasive breast cancer were enrolled. Patients from Center A were assigned to the training cohort (n = 184) and internal validation cohort (n = 81), whereas patients from Center B constituted an independent external test cohort (n = 71). The baseline clinicopathologic characteristics of the three cohorts are summarized in **Table 1**.

**Table 1:** Clinicopathologic characteristics of patients.

Characteristics	Training (n=184)	Validation (n=81)	Test (n=71)	P value
Age (year)	49.0 (41.0–55.2)	48.0 (39.0–54.0)	49.0 (39.0–55.0)	0.403
Menopause status (%)				0.507
Non-menopausal	89 (48.4)	45 (55.6)	38 (53.5)	
Post-menopausal	95 (51.6)	36 (44.4)	33 (46.5)	
HR status (%)				0.484
negative	80 (43.5)	33 (40.7)	25 (35.2)	
positive	104 (56.5)	48 (59.3)	46 (64.8)	
HER2 status (%)				0.757
negative	111 (60.3)	45 (55.6)	41 (57.7)	
positive	73 (39.7)	36 (44.4)	30 (42.3)	
KI67 status (%)				0.450
negative	32 (17.4)	12 (14.8)	16 (22.5)	
positive	152 (82.6)	69 (85.2)	55 (77.5)	
Molecular subtype (%)				0.818
HR+/HER2-	71 (38.6)	32 (39.5)	31 (43.7)	
HR+/HER2+	33 (17.9)	16 (19.8)	15 (21.1)	
HR-/HER2+	40 (21.7)	20 (24.7)	15 (21.1)	
HR-/HER2-	40 (21.7)	13 (16.0)	10 (14.1)	
Clinical T stage (%)				0.632
T1	32 (17.4)	20 (24.7)	18 (25.4)	
T2	104 (56.5)	42 (51.9)	35 (49.3)	
T3	26 (14.1)	9 (11.1)	7 (9.9)	
T4	22 (12.0)	10 (12.3)	11 (15.5)	
Axillary status (%)				0.994
apCR	79 (42.9)	35 (43.2)	31 (43.7)	
non-apCR	105 (57.1)	46 (56.8)	40 (56.3)	

apCR, axillary pathological complete response; ER, estrogen receptor; PR, progesterone receptor; HER2, human epidermal growth factor receptor; TNBC, triple-negative breast cancer.

The median age did not differ significantly among the training, internal validation, and external test cohorts (P = 0.403). No significant differences were observed in menopausal status, HR, HER2, Ki-67, molecular subtype, or clinical T stage across the three cohorts (all P > 0.05).

### 3.2 Independent clinical predictors of apCR in the training cohort

In the training cohort, univariable logistic regression showed that HR status was significantly associated with apCR, whereas HER2 status did not reach statistical significance. Nevertheless, HER2 status had been prespecified as an *a priori* clinically relevant covariate and was therefore retained in the multivariable model. After multivariable adjustment, both HR status and HER2 status were independently associated with apCR. Detailed results of the univariable and multivariable analyses are summarized in **Table 2**.

**Table 2:** Univariable and multivariable logistic regression analysis of characteristics associated with axillary pathologic complete response in the training cohort.

	Univariable analysis		Multivariable analysis	
Characteristics	Odds ratio	P value	Odds ratio	P value
Age (year)	0.995 (0.989,1.001)	0.082	1.004 (0.989,1.02)	0.602
Menopause status				
Non-menopausal				
Post-menopausal	0.696 (0.463,1.048)	0.083	0.795 (0.419,1.508)	0.482
HR status				
negative				
positive	0.486 (0.322,0.732)	0.001	0.363 (0.229,0.577)	**<0.001**
HER2 status				
negative				
positive	1.433 (0.899,2.285)	0.130	2.313 (1.359,3.937)	**0.002**
KI67 status				
negative				
positive	0.727 (0.527,1.004)	0.053	0.706 (0.421,1.183)	0.186
Molecular subtypes (%)				
HR+/HER2-				
HR+/HER2+	0.833 (0.42,1.653)	0.602		
HR-/HER2+	2.333 (1.187,4.588)	0.014		
HR-/HER2-	0.739 (0.395,1.383)	0.345		
Clinical T stage				
T1				
T2	0.891 (0.606,1.309)	0.557		
T3	0.444 (0.193,1.022)	0.056		
T4	1.2 (0.518,2.777)	0.670		

Variables with P < 0.05 in the multivariable analysis are highlighted in bold. apCR, axillary pathological complete response; HR, hormone receptor; HER2, human epidermal growth factor receptor.

### 3.3 Feature extraction and selection

Based on enhancement heterogeneity on DCE-MRI, tumors were partitioned into five spatially distinct habitat subregions using the Calinski–Harabasz index. A total of 5,985 habitat-based radiomic features were extracted from these subregions, whereas 1,197 whole-tumor radiomic features were derived from the corresponding DCE-MRI sequence. Following feature selection, 16 whole-tumor radiomic features and 20 habitat-based radiomic features were retained for subsequent model development.

### 3.4 Performance of models in predicting apCR

**Figure 3:**
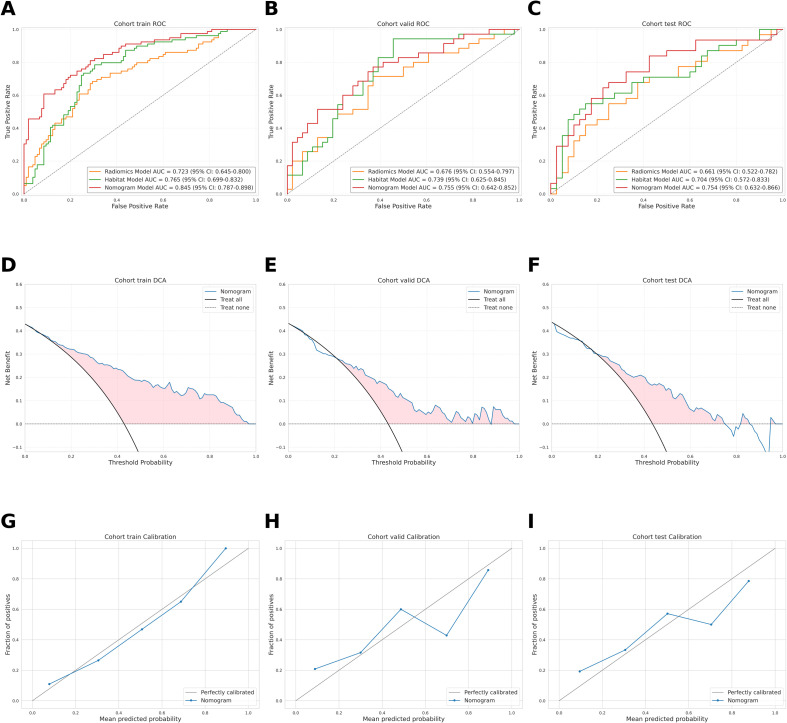
Receiver operating characteristic (ROC) curves of the radiomics, habitat, and nomogram models in the training cohort **(A)**, validation cohort **(B)**, and test cohort **(C)**. Decision curve analysis of the nomogram model in the training cohort **(D)**, validation cohort **(E)**, and test cohort **(F)**. Calibration curves of the nomogram model in the training cohort **(G)**, validation cohort **(H)**, and test cohort **(I)**. In the calibration plots, curves closer to the diagonal line, which represents ideal calibration, indicate better agreement between predicted and observed probabilities.

**Figure 4:**
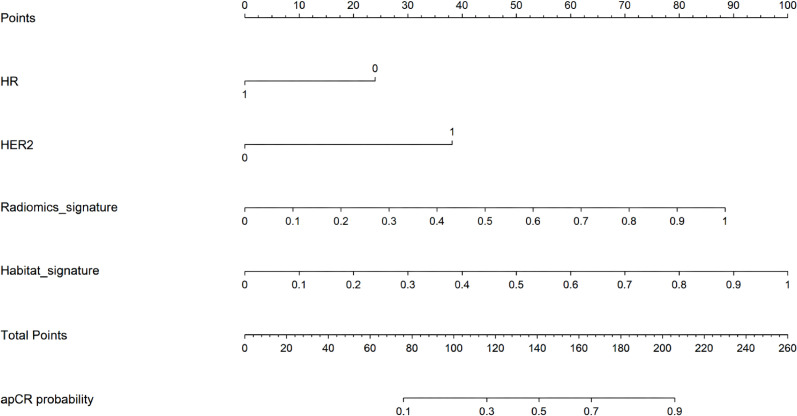
Nomogram for preoperative prediction of axillary pathological complete response (apCR) after neoadjuvant therapy. The nomogram integrates hormone receptor (HR) status, human epidermal growth factor receptor 2 (HER2) status, radiomics signature, and habitat signature. For an individual patient, points are assigned for each variable according to its value, and the sum of points corresponds to the estimated probability of apCR.

Compared with the radiomics model, the habitat model showed consistently higher AUC values across the training (0.765 vs. 0.723), internal validation (0.739 vs. 0.676), and independent external test cohorts (0.704 vs. 0.661) (**Table 3**, **Figures 3A–C**). The nomogram demonstrated the highest discriminative performance, with AUCs of 0.845, 0.755, and 0.754 in the training, internal validation, and independent external test cohorts, respectively (**Table 3**, **Figure 4**). Pairwise DeLong test results with Bonferroni correction are summarized in **Supplementary Table 4**. After correction, a significant difference was observed only between the nomogram and radiomics models in the training cohort (P = 0.0003), whereas all other pairwise comparisons were not statistically significant. Bootstrap-based optimism correction in the training cohort further showed that the optimism-corrected AUCs were 0.656 for the radiomics model, 0.680 for the habitat model, and 0.832 for the nomogram. The corresponding apparent AUCs and mean optimism values are also provided in **Supplementary Table 5**.

**Table 3:** Performance of three models in predicting pathologic complete response to neoadjuvant therapy in the training, validation and test cohorts.

Models and Cohorts	AUC (95%CI)	Accuracy (%)	Sensitivity (%)	Specificity (%)	PPV (%)	NPV (%)
Training						
Radiomics	0.723 (0.65,0.796)	0.696 (0.625,0.761)	0.684 (0.576,0.788)	0.705 (0.615,0.792)	0.635 (0.536,0.736)	0.747 (0.654,0.829)
Habitat	0.765 (0.695,0.832)	0.734 (0.668,0.799)	0.785 (0.69,0.868)	0.695 (0.605,0.782)	0.660 (0.563,0.756)	0.811 (0.728,0.89)
Nomogram	0.845 (0.789,0.901)	0.755 (0.687,0.816)	0.734 (0.623,0.827)	0.771 (0.679,0.848)	0.707 (0.596,0.803)	0.794 (0.703,0.868)
Validation						
Radiomics	0.676 (0.55,0.791)	0.617 (0.506,0.728)	0.571 (0.412,0.735)	0.652 (0.512,0.792)	0.556 (0.385,0.727)	0.667 (0.529,0.804)
Habitat	0.739 (0.623,0.84)	0.679 (0.568,0.778)	0.686 (0.528,0.839)	0.674 (0.537,0.808)	0.615 (0.455,0.769)	0.738 (0.609,0.87)
Nomogram	0.755 (0.648,0.861)	0.679 (0.566,0.778)	0.571 (0.394,0.737)	0.761 (0.612,0.874)	0.645 (0.454,0.808)	0.700 (0.554,0.821)
Testing						
Radiomics	0.661 (0.533,0.788)	0.634 (0.521,0.746)	0.548 (0.371,0.722)	0.700 (0.553,0.839)	0.586 (0.407,0.774)	0.667 (0.526,0.800)
Habitat	0.704 (0.569,0.825)	0.634 (0.521,0.746)	0.677 (0.5,0.833)	0.600 (0.439,0.75)	0.568 (0.409,0.725)	0.706 (0.545,0.848)
Nomogram	0.754 (0.637,0.871)	0.704 (0.584,0.807)	0.645 (0.454,0.808)	0.750 (0.588,0.873)	0.667 (0.472,0.827)	0.732 (0.571,0.858)

AUC, area under the curve; CI, confidence interval; PPV, positive predictive value; NPV, negative predictive value.

Decision curve analysis further indicated that the nomogram yielded greater net clinical benefit than the radiomics and habitat models across a range of clinically relevant threshold probabilities (**Figures 3D–F**). Calibration curves showed good agreement between predicted and observed apCR probabilities across all cohorts (**Figures 3G–I**).

**Figure 5:**
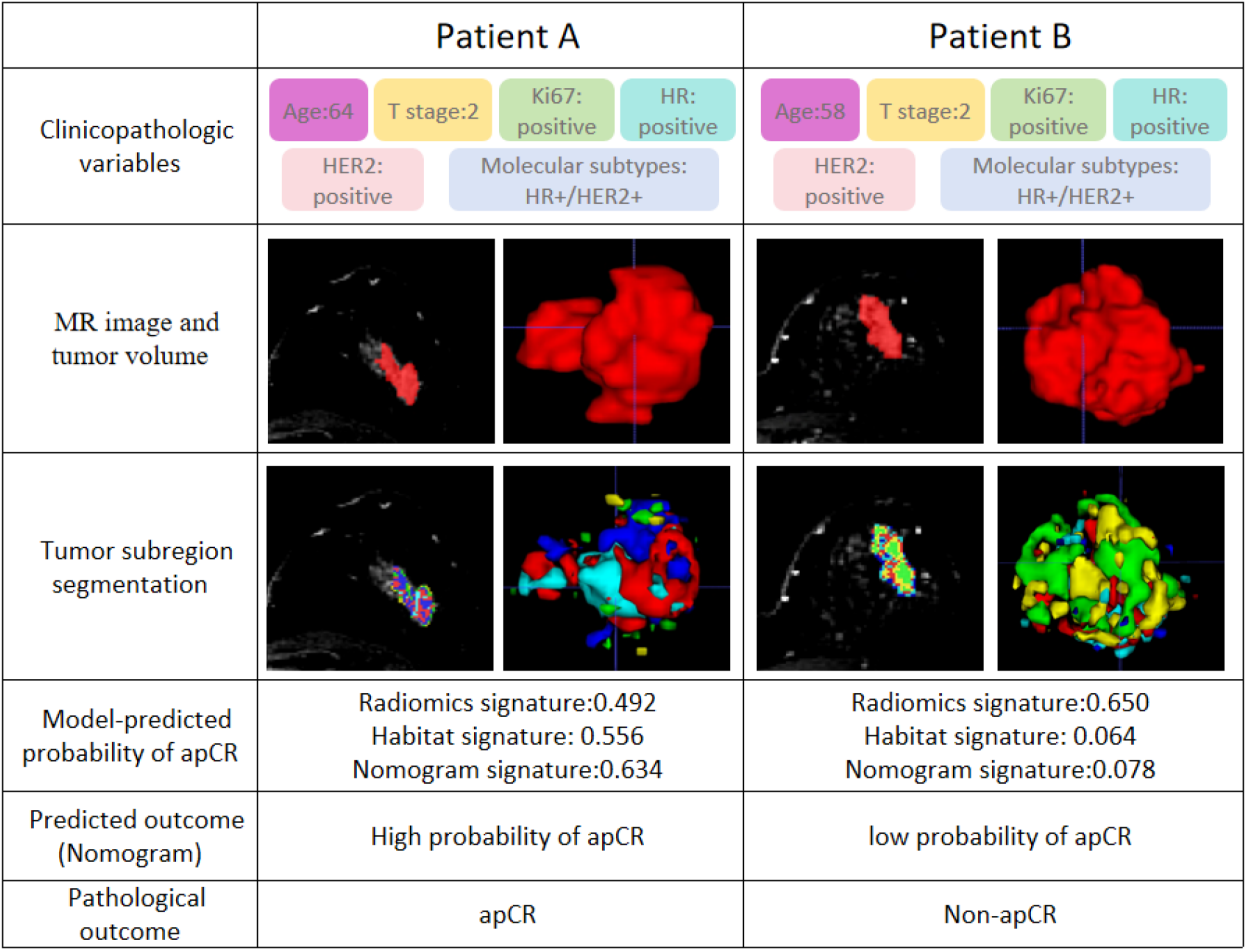
Representative cases illustrating the potential clinical application of the nomogram for predicting axillary pathological complete response (apCR) after neoadjuvant therapy (NAT). The two patients had similar clinicopathologic characteristics, including positive Ki-67 status, clinical T2 stage, positive hormone receptor (HR) status, positive human epidermal growth factor receptor 2 (HER2) status, but differed in age (64 vs. 58 years). Axial DCE-MRI images and corresponding habitat-based heterogeneity maps are shown. Compared with Patient **(A)**, Patient **(B)** exhibited more pronounced intratumoral spatial heterogeneity. The nomogram predicted an apCR probability of 63.4% for Patient **(A)** and 7.8% for Patient **(B)** Postoperative pathology confirmed apCR in Patient A but not in Patient **(B)** apCR, axillary pathological complete response; ER, estrogen receptor; PR, progesterone receptor; HER2, human epidermal growth factor receptor; HR, hormone receptor.

Representative cases of the nomogram are shown in **Figure 5**. Despite broadly similar clinicopathologic characteristics, the two patients demonstrated different patterns of habitat heterogeneity on DCE-MRI and corresponding predicted probabilities of apCR. The patient with more pronounced intratumoral heterogeneity showed a lower predicted probability of apCR, and the postoperative pathological findings were consistent with the prediction.

## 4 Discussion

Accurate preoperative assessment of axillary response after NAT has become increasingly important in the management of node-positive breast cancer (7). Reliable identification of apCR may allow selected patients to avoid unnecessary axillary lymph node dissection (24). In this multicenter study, a habitat-based radiomics model demonstrated favorable performance for predicting apCR across the training, internal validation, and independent external test cohorts. Notably, the highest predictive performance was achieved when habitat-derived heterogeneity features were combined with whole-tumor radiomic features and clinicopathologic variables, resulting in a nomogram with improved discrimination across cohorts.

Clinical responses to NAT in breast cancer are heterogeneous, and conventional clinicopathologic factors explain this variability only in part (25). In our study, multivariable analysis identified both HR status and HER2 status as independent predictors of apCR, consistent with their established associations with treatment sensitivity (26–28). Although HER2 status was not significant in univariable analysis, it had been prespecified as a clinically relevant covariate and was therefore retained in the multivariable model, where it emerged as an independent predictor after adjustment. This finding underscores the importance of multivariable modeling for estimating the independent contribution of clinically relevant factors. However, clinicopathologic variables alone did not fully account for the observed response heterogeneity, suggesting that additional sources of biological variability may also be relevant and supporting the use of imaging-based analyses to capture complementary tumor phenotypes (29). In this context, MRI-based radiomics has been explored to complement clinicopathologic assessment of treatment response, but most approaches rely on whole-tumor features that average spatially heterogeneous treatment effects (14–16, 30, 31). In our study, the whole-tumor radiomics model showed relatively modest predictive performance, suggesting that global feature aggregation may be insufficient in the neoadjuvant setting.

Habitat radiomics, in contrast, focuses on modeling intratumoral spatial heterogeneity rather than treating the tumor as a uniform entity (32, 33). In this study, the habitat-based model consistently outperformed the whole-tumor radiomics model across the training, internal validation, and independent external test cohorts, with higher AUCs observed in all three cohorts (0.765 vs. 0.723, 0.739 vs. 0.676, and 0.704 vs. 0.661, respectively). Moreover, incorporation of the habitat signature into the combined nomogram further improved discriminative performance, yielding the highest AUCs across cohorts. These findings indicate that habitat-derived features capture complementary information beyond global radiomic descriptors and clinicopathologic factors. This added value likely reflects spatially heterogeneous tumor characteristics, such as regional perfusion differences and therapy-induced changes that coexist within tumors after neoadjuvant treatment and produce heterogeneous enhancement on DCE- MRI (23, 34).

Although several previous studies have attempted to incorporate imaging features from ALNs to predict axillary response after NAT (15, 35, 36), this approach remains difficult to implement in routine practice. Breast MRI often provides incomplete coverage of the axillary region (37), and its spatial resolution may be insufficient for reliable visualization and delineation of metastatic ALNs. Moreover, when multiple lymph nodes are involved, it is often difficult to establish an exact correspondence between individual nodes identified on imaging and those sampled pathologically (38). This may introduce uncertainty in label assignment and, in turn, compromise model training and interpretation. For these reasons, we adopted a more reproducible and clinically applicable strategy by focusing on the primary tumor ROI. Compared with direct ALN delineation, the primary lesion is usually better depicted on breast MRI and can be segmented more consistently, thereby reducing feature instability related to segmentation variability. At the same time, the imaging phenotype of the primary tumor may partly reflect tumor heterogeneity, vascular characteristics, microenvironmental properties, and treatment sensitivity (32, 39), all of which may also be related to regional therapeutic response. In this context, primary tumor habitat features may serve as an indirect imaging surrogate for axillary response after NAT.

Beyond its predictive performance, the proposed habitat-based framework also offers practical advantages for clinical implementation. Whereas many prior radiomics studies have relied on multiparametric MRI protocols (40–42), our approach was based on a single, routinely acquired DCE-MRI sequence, emphasizing feasibility and reproducibility in real-world settings. Although multiparametric imaging can provide complementary structural or cellular information, it also entails increased acquisition complexity and greater susceptibility to inter-scanner and inter-protocol variability (43). By leveraging a standardized single-sequence strategy, our framework may facilitate broader clinical adoption and improve robustness across institutions.

Recent breast MRI studies have suggested that habitat-based analysis may improve the assessment of treatment response by capturing intratumoral heterogeneity. Ji et al. (44) reported a single-center pretreatment DCE-MRI habitat-radiomics model in which voxel-wise clustering was used to define three habitat subregions, and the most informative subregion was subsequently incorporated with clinicopathologic variables into a nomogram. Our study extends this line of research in two respects. First, it was performed in a multicenter setting and included an independent external test cohort, allowing a more stringent evaluation of model generalizability. Second, rather than focusing on a single optimal habitat subregion, we developed both whole-tumor radiomics and habitat-based models and further integrated these imaging signatures with clinicopathologic variables into a combined nomogram. Taken together, our findings support the potential added value of habitat-derived imaging information for predicting treatment response in a more heterogeneous clinical setting.

A decrease in model performance from the training cohort to the internal validation and independent external test cohorts was observed. This is not unexpected and may be related to model fitting in the training cohort, the relatively limited sample sizes of the validation and test cohorts, and between-center heterogeneity in MRI acquisition protocols and treatment strategies. Notwithstanding this decline, the nomogram consistently achieved the highest AUC across cohorts, suggesting a stable trend towards improved discrimination. Its incremental value, however, still warrants confirmation in larger prospective multicenter studies.

This study has several limitations. First, its retrospective design may have introduced selection bias, and the limited representation of some clinically relevant subgroups may have affected model stability and subgroup-specific performance. Second, although image preprocessing was performed, scanner- and protocol-related variability was not formally harmonized and may still have influenced feature robustness and model transferability. Third, the model was developed using pretreatment imaging features derived from the primary tumor only and did not incorporate direct imaging information from metastatic axillary lymph nodes, which may have limited its ability to fully capture axillary response after NAT. Finally, we did not systematically compare multiple machine-learning classifiers, and the clinical utility of the proposed nomogram still requires confirmation in larger prospective multicenter cohorts.

## 5 Conclusion

In conclusion, this multicenter study suggests that the proposed nomogram may offer a noninvasive means of estimating the likelihood of apCR after NAT in node-positive breast cancer. The addition of habitat-derived features to conventional radiomics and clinicopathologic variables was associated with improved predictive performance. However, further validation in larger prospective multicenter cohorts is required before clinical implementation.

## Data Availability

The raw data supporting the conclusions of this article will be made available by the authors, without undue reservation.
