# Supplementary material for: MRI-based habitat radiomics for preoperative prediction of axillary pathological complete response in breast cancer after neoadjuvant therapy: a multicenter study
Source: Front Oncol. 2026 May 13;16:1801530. doi: 10.3389/fonc.2026.1801530 (PMC13212183; doi:10.3389/fonc.2026.1801530)
Supplement: Supplementary file 1 [file Table1.docx]

Supplementary Material

# S1. Histopathological examination

Baseline clinical data, including age, menstrual status, and other relevant characteristics, was obtained from the hospital’s electronic medical records. The expression status of estrogen receptor (ER), progesterone receptor (PR), human epidermal growth factor receptor 2 (HER2), and the Ki-67 index were determined via immunohistochemistry (IHC). Hormone receptor (HR) positivity was defined as ≥1% nuclear staining of either ER or PR; and tumors were classified as HER2-negative if IHC staining was 0 or 1+, and as HER2-positive if the IHC score was 3+. In cases with an IHC score of 2+, HER2 status was determined by fluorescence in situ hybridization to assess gene amplification. Based on receptor status, tumors were classified into four molecular subtypes: (i) HR+/HER2-, (ii) HR+/HER2+, (iii) HR-/HER2-, and (iv)HR-/HER2-. Ki-67 expression was evaluated using a cutoff value of 20%; tumors with <20% staining were considered to have low proliferation, while those with ≥20% were considered highly proliferative. Clinical tumor stage was assessed according to the American Joint Committee on Cancer TNM staging system manual, 8th edition.

# S2. MRI Acquisition Parameters

All patients underwent dynamic contrast-enhanced T1-weighted MRI with fat suppression using dedicated bilateral breast coils. MRI examinations were performed in the prone position on 3.0-T scanners across both participating centers. Imaging was conducted using systems from GE Healthcare, Siemens Healthineers, United Imaging Healthcare, and Philips Healthcare. For DCE-MRI, a fat-suppressed T1-weighted precontrast acquisition was obtained, followed by multiphase postcontrast imaging using a three-dimensional gradient-echo sequence. A gadolinium-based contrast agent was administered intravenously using an MRI-compatible power injector at a weight-based dose (0.2 mmol/kg) and a standardized injection rate, followed by a saline flush. Multiple postcontrast phases were acquired at regular intervals after contrast administration. The detailed MRI acquisition parameters of the two participating centers are listed in Table 1.

For radiomic analysis and tumor segmentation, early postcontrast images acquired approximately 90–120 seconds after contrast injection were selected, as they provide optimal lesion-to-background contrast. Across both centers, MRI examinations were performed according to routine clinical protocols, and standardized image preprocessing was applied to minimize inter-scanner and inter-protocol variability.

# S3. Determination of the Number of Habitat Subregions

To determine the optimal number of habitat subregions, k-means clustering was performed with k ranging from 2 to 5. The Calinski–Harabasz index was calculated for each clustering solution, and k = 5, corresponding to the highest index value, was selected for subsequent habitat definition. The Calinski–Harabasz index values for the different k values are listed in Table 2.

**Supplementary Tables**

Table 1. MR scanning parameters for the patients in two centers

| **Center** | **Scanner** | **Sequence** | **TR** | **TE** | **Slice thickness (mm)** | **Matrix** | **FOV (mm)** | **Slice Gap (mm)** | **Flip Angle** |
| --- | --- | --- | --- | --- | --- | --- | --- | --- | --- |
| **A** | **GE 3.0T** | **T1+C** | 4.4 | 2.1 | 1.8 | 384x 320 | 360x360 | -0.8 | 10 |
| **A** | **uMR 790 3.0T** | **T1+C** | 5.0 | 2.18 | 1.2 | 288x 288 | 340x340 | -0.4 | 10 |
| **A** | **Siemens 3.0T** | **T1+C** | 4.6 | 1.63 | 1.6 | 416x312 | 360x360 | -0.48 | 10 |
| **B** | **Philips 3.0T** | **T1+C** | 4.1 | 2.0 | 2.4 | 228x319 | 256x350 | -1.2 | 12 |

Table 2 Calinski–Harabasz index values for different candidate numbers of habitat subregions (k = 2–5).

| k | 2 | 3 | 4 | 5 |
| --- | --- | --- | --- | --- |
| Calinski–Harabasz index | 176381.09 | 214070.40 | 250004.05 | 285653.93 |

Table 3. Number of features retained after each selection step

| Feature type | Original features | After ICC | After Mann–Whitney U test | After mRMR | After LASSO | Final selected |
| --- | --- | --- | --- | --- | --- | --- |
| Radiomics | 1197 | 611 | 78 | 50 | 15 | 15 |
| Habitat | 5985 | 1302 | 107 | 50 | 16 | 16 |

Table 4. Pairwise DeLong test results for comparison of ROC curves among the radiomics, habitat, and nomogram models

| **Cohort** | **Comparison** | **Z value** | **P value** | **Significant after Bonferroni correction** |
| --- | --- | --- | --- | --- |
| Training |  |  |  |  |
|  | Radiomics vs Habitat | 0.8755 | 0.3813 | No |
|  | Radiomics vs Nomogram | 3.6064 | ***0.0003*** | Yes |
|  | Habitat vs Nomogram | 2.1888 | 0.0286 | No |
| Validation |  |  |  |  |
|  | Radiomics vs Habitat | 0.7885 | 0.4304 | No |
|  | Radiomics vs Nomogram | 1.5336 | 0.1251 | No |
|  | Habitat vs Nomogram | 0.2592 | 0.7955 | No |
| Test |  |  |  |  |
|  | Radiomics vs Habitat | 0.5129 | 0.6080 | No |
|  | Radiomics vs Nomogram | 1.9518 | 0.0510 | No |
|  | Habitat vs Nomogram | 0.7895 | 0.4298 | No |

Table 5. Internal bootstrap validation: optimism-corrected AUC

| Model | apparent AUC | mean optimism | optimism-corrected AUC | bootstrap iterations |
| --- | --- | --- | --- | --- |
| Radiomics | 0.723 | 0.067 | 0.656 | 1000 |
| Habitat | 0.765 | 0.085 | 0.680 | 1000 |
| Nomogram | 0.845 | 0.013 | 0.832 | 1000 |
